# Exploring the analytical power of the QTOF MS platform to assess monoclonal antibodies quality attributes

**DOI:** 10.1371/journal.pone.0219156

**Published:** 2019-07-10

**Authors:** Ricardo A. Gomes, Conceição Almeida, Catarina Correia, Ana Guerreiro, Ana Luísa Simplício, Isabel A. Abreu, Patrícia Gomes Alves

**Affiliations:** UniMS – Mass Spectrometry Unit, ITQB/IBET, Oeiras, Portugal; Fisheries and Oceans Canada, CANADA

## Abstract

The biopharmaceutical industry is growing at a fast pace, making nowadays 20% of the pharma market. Within this market, therapeutic monoclonal antibodies (mAbs) are the dominant product class. With the patent expirations, biosimilars and, perhaps more relevant, biobetters, are in fast development. Thus, a comprehensive characterization at the molecular level of antibodies heterogeneity such as glycoforms, post-translational modifications (PTMs) and sequence variations is of utmost importance. Mass spectrometry (MS)-based approaches are undoubtedly the most powerful analytical strategies to monitor and define an array of critical quality attributes on mAbs. In this work, we demonstrate the analytical power of the Q-TOF MS platform for comprehensive and detailed analysis at molecular levels of an in-house produced mAb. This methodology involves minimal sample preparation procedures and provides an extensive collection of valuable data in a short period of time.

## Introduction

At present, monoclonal antibodies (mAbs) are used to treat several clinical conditions such as cancer, metabolic disorders and autoimmune diseases[1,2]. mAbs are the most important product class in the biopharmaceutical industry[3], being produced by recombinant protein technology. The clinical demands for new therapies and for products that meet safety and efficiency requirements have placed increasing pressure on the development and manufacturing processes[4]. Both processes must be highly productive and cost-effective. To meet this demand, analytical assays are critical, especially when dealing with such complex entities as mAbs.

An in-depth characterization of mAbs is of utmost importance not only before they can be used in clinical trials but also during product development stages. PTMs including oxidation, deamidation, glycosylation, glycation, proteolysis, and others, may significantly alter the efficacy and safety of the protein drug [5–9]. Thus, regulatory agencies require a full characterization of the structural, biological and chemical heterogeneity of any biological drug product[10]. mAbs characterization is analytical challenging as these products are inherently heterogeneous, due to the presence of PTMs, incomplete processing, susceptibility to degradation and disulphide shuffling [6,11–13].

Ideally, the methodologies applied to monitor the final product quality should also be applied throughout the entire pipeline to provide qualitative and quantitative information. This will enable optimization of parameters/operations in different stages of the protein drug lifecycle, from clone selection and product development to quality control and drug disposition. To comply with these requests, the analytical methods employed must provide detailed information, and ideally should be fast and with little sample manipulation.

An array of methods and separation techniques are used to monitor intact antibody process consistency and identify product variants and impurities. These include capillary electrophoresis and chromatography methodologies as ion exchange, reversed-phase, size-exclusion or hydrophobic-interaction[13]. These methods are time-consuming, require several instrumental setups and specific sample preparation methodology. These features, in combination with the high complexity of biopharmaceuticals, makes direct and fast measures of product quality in real time very challenging. Additionally, these methods are not able to monitor product quality attributes at the molecular level (e.g., detect the site where a PTM is occurring). In this context, mass spectrometry is emerging as the most powerful analytical tools available. MS has been a widely used as the tool of choice for protein structure characterization as well as for the analysis of complex samples (as proteomics). The selectivity, specificity, sensitivity, dynamic range, mass accuracy, and resolution of modern MS instrumentation, as well as the ability to be coupled with different modes of separation, make this analytical tool invaluable for qualitative and quantitative analysis of biologics[14,15]. Combining powerful and robust separation technologies with exceptional selectivity, based on precise mass accuracy and high resolution, MS can provide a full analysis of multiple components and multiple attributes of a heterogeneous biomolecule in a single assay using a single instrument platform[14,16]. Thus, MS can provide fast and direct measure of product quality attributes in complex samples and this would be very beneficial for the development and manufacture of complex biologics. Several MS-based workflows have been used to monitor mAbs molecular critical quality attributes[17–19]. One example of the advantages in using MS-based methodologies is the glycans mapping. The gold standard and widely used procedure is the analysis of free N-glycans labelled with a fluorescence compound by HILLIC chromatography or capillary electrophoresis[19]. While this approach is sensitive and precise it is quite time consuming and requires the protein purification prior to the glycan analysis. The amount of time required for sample preparation, the amount of protein needed for the analysis and the length of the chromatography make it difficult to implement this method for a real time fast profiling during the mAbs production. By contrast, the use of MS workflows provides a fast glycan profiling with high sensitivity. Despite these advantages, challenges with sample preparation and data processing remains. With the huge amount of data generated in a single MS experiment, data processing and analysis can be a bottleneck.

This work aimed to implement a comprehensive and fast mAb characterization with minimal sample preparation procedures, without any specific method optimization. Using the TripleTOF 6600 system four levels of characterization were performed: (1) protein level analysis of intact mAb; (2) protein level analysis of reduced mAb; (3) peptide mapping and (4) N-free glycan analysis. This work highlights the perfect combination of analytical power, sample preparation simplicity, and fast data assembly.

## Material and methods

### Chemicals

The monoclonal antibody sample was kindly supplied by Dr. Ana Barbas from iBET, Portugal. LC-MS grade acetonitrile 0.1% formic acid and LC-MS grade water 0.1% formic acid were acquired from Fisher Chemicals (Geel, Belgium). Graphite Tip-columns were acquired from Thermo Fisher Scientific (Waltham, USA); Sequencing Grade Modified Trypsin was purchased from Promega (Madison, USA); DTT, iodoacetamide, ammonium bicarbonate and PNGase F from Sigma (St Louis, USA); 10 kDa and 30 kDa amicon filters units from Merck Millipore (Cork, Ireland).

Tuning solution and beta-galactosidase digest standard were obtained from Sciex (Framinghan, USA). The glycan standard asialo diantennary plus proximal α1→6Fucose (0N-2A+F) and asialo diantennary (0N-2A) were obtained from Theraproteins (Oeiras, Portugal).

### Sample preparation

Ten micrograms of the monoclonal antibody was desalted in 30 kDa Amicon filters units (Millipore) using 10% acetonitrile in 0.1% formic acid. The desalting procedure was repeated 10 times, according to the manufacturer’s instructions. Briefly, the amicon filter was pre-rinsed with 400 μL of 0.1% formic acid and spin at 12,000 x g for 7 minutes. The antibody was diluted to 400 μL of 10% acetonitrile in 0.1% formic acid, added to the amicon device and spin at 12000 x g for 7 minutes. This procedure was repeated 10 times. The desalted antibody solution was recovered and directly analysed by LC-MS as described in the LC-MS and LC-MSMS analysis section.

For the analysis of the deglycosylated antibody a deglycosylation step using PNGase enzyme was performed. Twenty micrograms of the monoclonal antibody was diluted with 20 mM Ammonium bicarbonate buffer and PNGase was added at a 1:15 W/W ratio. The incubation was performed overnight at 37 °C. The deglycosylated antibody was purified using 30 kDa Amicon filters following the procedure described above.

The free N-glycans was recovered in the flow through of the first amicon centrifugation. The N-glycans were purified using graphite tip-columns, according to the manufacturer’s instructions. Briefly, hypercarb tip was conditioned with the releasing solution (30% acetonitrile in 0.1% formic acid) and washed three times with 0.1% formic acid in LC-MS water. N-glycans sample was allowed to bind to the column by aspirating/expelling the samples 40 times. Sample was washed with 0.1% formic acid in LC-MS water and N-glycans were eluted using 30% acetonitrile in 0.1% formic acid[20]. Purified N-glycans were dried and resuspended in LC-MS water before MS analysis.

For the protein level analysis of reduced antibody (heavy and light chain analysis), 20μg of antibody was diluted in 50 mM ammonium bicarbonate buffer and reduced with 10 mM DTT for 40 min at 56 °C. Prior to LC-MS analysis, the reduced antibody was purified using 10 kDa amicon filters units (Millipore) following the procedure described above.

For peptide analysis, 20 μg of antibody was reduced with 10 mM DTT for 40 min at 56 °C, followed by alkylation with 55 mM iodoacetamide for 30 min in the dark. Excessive iodoacetamide was quenched by further incubation with 10 mM DTT for 10 min in the dark. The resulting sample was digested with trypsin at a 1:50 W/W protein/trypsin ratio overnight at 37 °C. Trypsin digestion was stopped by the addition of 2% formic acid. The sample was dried and resuspended in LC-MS water 0.1% formic acid prior to LC-MS analysis.

### LC-MS and LC-MSMS analysis

All analyses were performed on a TripleTOF 6600 mass spectrometer (Sciex) coupled with NanoLC system (Eksigent NanoLC 400 system).

For protein level analysis (intact or reduced mAb), samples were analysed by MicroLC-MS using an Eksigent HALO Fused-Core C18, 90 Å, 2.7 μm, 50 x 0.5 mm ID. The following LC conditions were used: flow rate 10 μL/min, column temperature 40 °C, mobile phase A contained water with 0.1% formic acid (LC-MS grade) and mobile phase B contained acetonitrile with 0.1% formic acid (LC-MS grade). The gradient was as followed: 5% B for 2 min; 5–60% B for 5 min; 60–95% B for 1 min; 95% B for 2 min; 95–5% B for 1 min; 5% B for 4 min. For intact antibody analysis, 50 to 200 ng were injected on-column, while for reduced antibody analysis 80 ng was loaded. The MS was set for intact protein mass mode with a TOF-MS scan for 1 sec from 400–4,000 m/z. The following ESI ionization parameters were used: ion source gas 1 set to 25, ion source gas 2 set to 0, curtain gas set to 35, ionspray voltage floating set to 5,500 V, ion source temperature set to 0, declustering potential set to 150 and collision energy set to 20. The MS was calibrated externally using the Tuning solution from Sciex.

For peptide analysis, 2 μg of tryptic-digest were used for information-dependent acquisition (IDA) analysis by MicroLC-MS. A reversed-phase microLC column Eksigent HALO Fused-Core C18, 90 Å, 2.7 μm, 150 x 0.5 mm ID was used. The following LC conditions were used: flow rate 5 μL/min, column temperature 30 °C, mobile phase A contained water with 0.1% formic acid (LC-MS grade) and mobile phase B contained acetonitrile with 0.1% formic acid (LC-MS grade). The gradient was as followed: 5% B for 1 min; 5–30% B for 55 min; 30–95% B for 3 min; 95% B for 3 min; 95–5% B for 2 min; 5% B for 6 min.

The mass spectrometer was set for IDA scanning full spectra (400–2,000 m/z) for 250 ms. The top 40 ions were selected for subsequent MSMS scans (150–1,800 m/z for 50 ms each) using a total cycle time of 2.3 s. The selection criteria for precursor ions included a charge state between +2 and +5 and counts above a minimum threshold of 125 counts per second. Ions were excluded from further MSMS analysis for 12 s. Fragmentation was performed using rolling collision energy with a collision energy spread of 5. The following ESI ionization parameters were used: ion source gas 1 set to 20, ion source gas 2 set to 0, curtain gas set to 35, ionspray voltage floating set to 5500 V, ion source temperature set to 0, declustering potential set to 80 and collision energy set to 10. MS was calibrated externally using an LC-MSMS analysis of a beta-galactosidase digest standard (from Sciex), with 200 fmol injected on-column.

Free N-glycans were analysed by NanoLC-MS with a trap and elution configuration using a Nano cHiPLC Trap column (200 μm × 0.5 mm Trap Graphitic Carbon 3 μm) and Nano column (75 μm × 15 cm Graphitic Carbon 3 μm 120 Å). Water with 0.1% formic acid (LC-MS grade, solvent A) and acetonitrile with 0.1% formic acid (LC-MS grade, solvent B) were used. The sample was loaded in the trap column at a flow rate of 2 μL/min for 10 min using 100% mobile phase A. Glycans separation was performed in the nano column at a flow rate of 300 nL/min. The gradient was as follows: 5% B for 1 min; 5–40% B for 15 min; 40–95% B for 2 min; 95% B for 7 min; 95–5% B for 1 min; 5% B for 19 min. The mass spectrometer was set for IDA scanning full spectra (400–2,000 m/z) for 250 ms. The top 20 ions were selected for subsequent MSMS scans (150–1,800 m/z for 100 ms each) using a total cycle time of 2.25 s. The selection criteria for precursors ions included a charge state between +2 and +4 and counts above a minimum threshold of 200 counts per second. Ions were excluded from further MSMS analysis for 12 s. Fragmentation was performed using collision energy of 25 with a collision energy spread of 5. The following ESI ionization parameters were used: Ion source gas 1 set to 15, Ion source gas 2 set to 0, curtain gas set to 30, ionspray voltage floating set to 2400 V, ion source temperature set to 100 °C, declustering potential set to 80 and collision energy set to 10. MS was calibrated externally using an LC-MSMS analysis of a two glycan standard mixture [asialo diantennary plus proximal α1→6Fucose (0N-2A+F) and asialo diantennary (0N-2A)].

### Data analysis

Data from the protein level analysis (intact or reduced mAb) was analysed using Sciex software packages PeakView version 2.2 with the BioTool Kit MicroApp, and BioPharmaView. The BioTool Kit MicroApp reconstruct protein setting was: start m/z of 1,000 and stop m/z of 4,000; output mass from 10,000–200,000 Da with a step mass of 1 Da and an input spectrum resolution of 2500. For the identification of the mAb proteoforms, we used the BioPharmaView software. The following parameters were used: 1) intact protein settings: peak threshold set to ≥ 5%, Gaussian-smoothing set to 0.90 points and the number of TOFMS spectra to combined set to 50 scans; 2) reconstruction processing settings: Iterations set to 20; signal to noise threshold set to ≥ 20; resolution set to 2,500; Gaussian-smoothing set to 0; relative protein results threshold set to ≥ 1%; 3) processing parameters: matching tolerance set to ± 5 Da.

For the relative quantification we used the peak areas of the reconstructed protein species with an intensity threshold > 1% retrieved by the PeakView software. The relative glycosylation percentage was defined as:

$$\frac{\sum Peak\;area\;of\;the\;reconsctructed\;glycoform}{\sum Peak\;area\;of\;all\;detected\;glycoforms}\;x\;100$$

The tryptic peptide data were analysed using the ProteinPilotsoftware, with the Paragon search engine (version 5.0, Sciex). The search was performed against a database containing the sequences of the proteins of interest and the following search parameters were set: Iodoacetamide, as Cys alkylation; Tryspsin, as digestion; TripleTOF 6600, as the Instrument; ID focus as biological modifications; search effort as thorough; and a FDR analysis. Only peptides with a 95% confidence were considered.

The number of modified peptides and the relative abundance (in percentage) of the detected mAb modification were calculated automatically by the ProteinPilotfull report. It computes the fraction of total ion signals having the modification of all forms of the same base peptide sequences, as measured via peptide elution apex intensities. Results from a representative experiment was presented.

Glycan analysis was performed using GlycoworkBench[21] and GlycoMod Tool from ExPASy. N glycans were identified by CID-MSMS data using the fragment ion matching for the low molecular weight oxonium ions m/z 204.08 (HexNAc^+^) and 366.11 (Hex-HexNAc^+^). The oxonium ion characteristic of sialic acid-containing glycan (292.09; NeuNAc^+^) were also performed. Monoisotopic masses were searched using the GlycoMod tool from ExPASy, considering a maximum of 10 ppm mass deviation. Assigned glycan compositions were confirmed by MSMS data using the GlycoworkBench software. Manual curation of MSMS results was also performed when needed. The XICs peak areas of the glycans m/z ions, retrieved by the PeakView software, was used to perform the relative quantification. The relative glycans percentage was defined as:

$$\frac{\sum XICs\;peak\;area\;of\;for\;glycan\frac{m}{z}ions}{\sum XICs\;peak\;area\;of\;all\;detected\;glycan\frac{m}{z}ions\;}\;x\;100$$

The use of XIC peak areas for the relative quantification have a shortcoming as ionization efficiencies may depend on individual structures. Differences in ionization efficiencies leads to differences in ion intensity. A more accurate relative quantification may be performed using internal standards. However, as reported by Rogers and co-workers[16], there is an excellent correlation between the glycosylation percentage determined by glycopeptide analysis (without the use of internal standards) and determined by the gold-standard HILLIC-LC for glycosylation profile.

## Results and discussion

**Fig 1:**
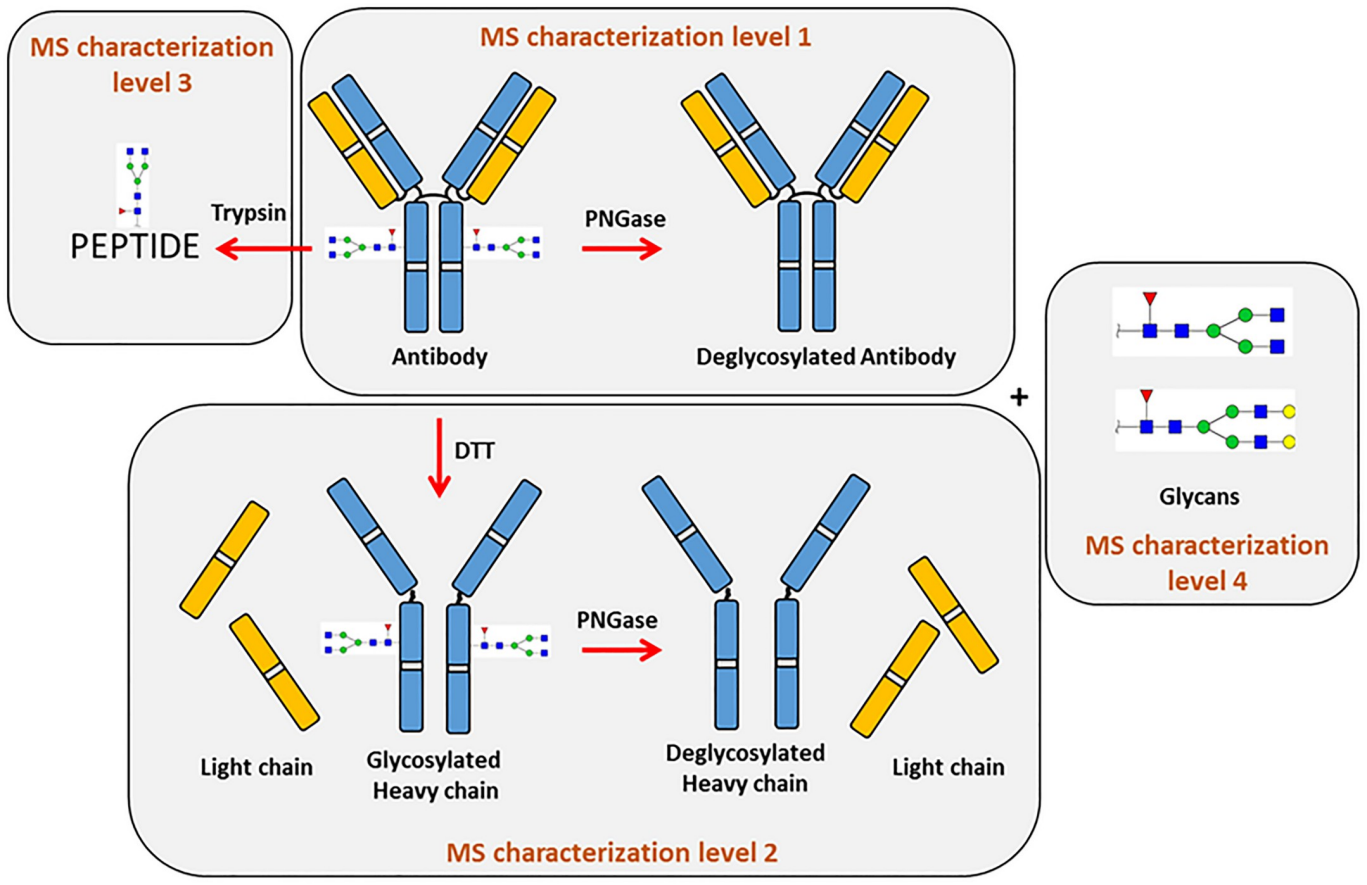
Schematic representation of the workflow used for mAbs analysis, highlighting the four characterization levels explored.

In this work, we describe a workflow for a comprehensive characterization of monoclonal antibodies using a single MS platform and minimal sample preparation procedures (Fig 1). Four characterization levels were explored: 1) protein level analysis with the mass measurement of intact and deglycosylated antibody for the detection of molecular isoforms; 2) protein level analysis with the mass measurement of reduced and reduced+deglycosylated antibody; 3) peptide level analysis of trypsin-digested antibody to characterize (glyco)peptides and unveiling PTMs; 4) free N-glycans analysis.

### 1) Protein level analysis of intact antibody

**Fig 2:**
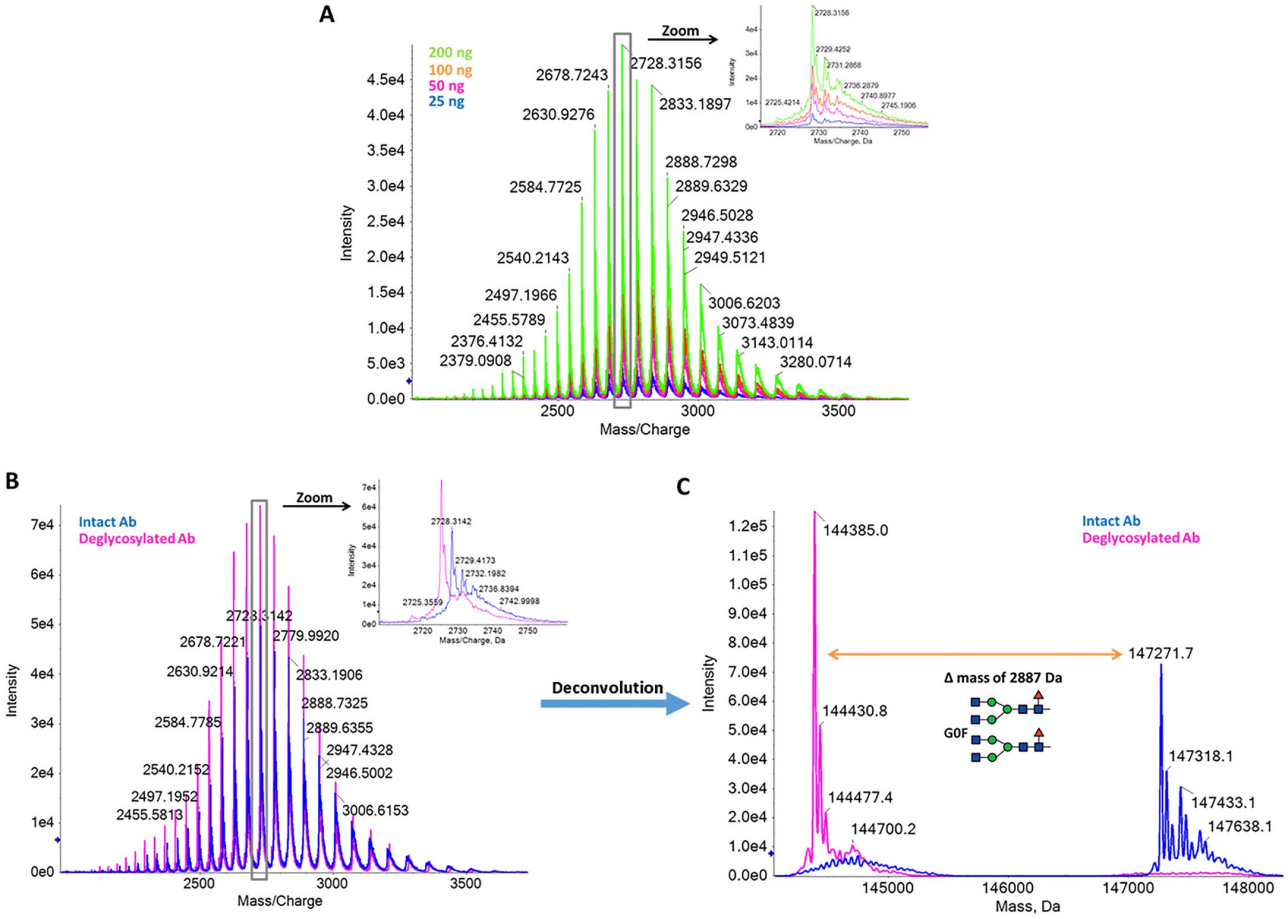
Protein level analysis of intact recombinant monoclonal antibody (characterization level 1). (A) TOF MS spectra of monoclonal recombinant antibody, from 25 ng to 200 ng on column injection. The inset shows a zoom view of the m/z 2728.31. (B) TOF MS spectra of untreated (blue line) and PNGase-treated (pink line) antibody. A mass shift and reduction of heterogeneity are observed upon PNGase treatment. (C) Deconvolution results of intact (blue line) and deglycosylated antibody (pink line). Glycosylation heterogeneity is clearly detected in the intact antibody. The two major glycosylation species detected are highlighted in the zoom view.

Full MS spectra of mAb are showed in Fig 2A. The mass spectrum shows the typical charge distribution observed for large proteins with MS resolution and MS data quality that enabled the detection of antibody heterogeneity (Fig 2A, inset). Spectra deconvolution allowed the assignment of different glycoforms, identified due to the typical mass difference of 162 Da (a hexose molecule). We tested a mAb amount injected on-column from 25–200 ng and obtained, for the most abundant glycoform, a mass value of 147,274 ± 2 Da (13 ppm). The rational of varying the amount of antibody injected was to determine the minimal mAb amount that should be used in order to detect the low abundance mAb proteoforms. Additionally, we used these different injections to determine the accuracy of the mass determination. To further characterize glycosylation heterogeneity and determine the main glycan structures present, we treated the antibody with PNGase-F (for N-glycans removal) prior to MS analysis (Fig 2B). We observed a clear reduction in mAb heterogeneity and a mass decrease. Considering the glycan structures commonly detected on mAbs and the mass difference observed (2887 Da, Fig 2C), we identified three major glycoforms (Fig 2C): G0F/G0F (16 ppm), G0F/G1F (4.5 ppm) and G0F/G2F or G1F/G1F (17 ppm). We detected two additional protein species in the range of 45–48 Da, for each glycoform. These mass differences are consistent with formic acid adducts.

Comparing to the theoretical antibody mass with the G0F glycan structure, we observed a mass difference of -37 Da. This is consistent with either an N-terminal glutamate modification to pyroglutamic acid in both light chains (mass difference of -36 Da) or an N-terminal glutamine modification to pyroglutamic acid in both heavy chains (mass difference of -34 Da). This ambiguity highlights the need to perform other analysis, namely the protein levels analysis of reduced antibody and peptide analysis to fully unveil possible PTMs.

### 2) Protein level analysis of reduced antibody

**Fig 3:**
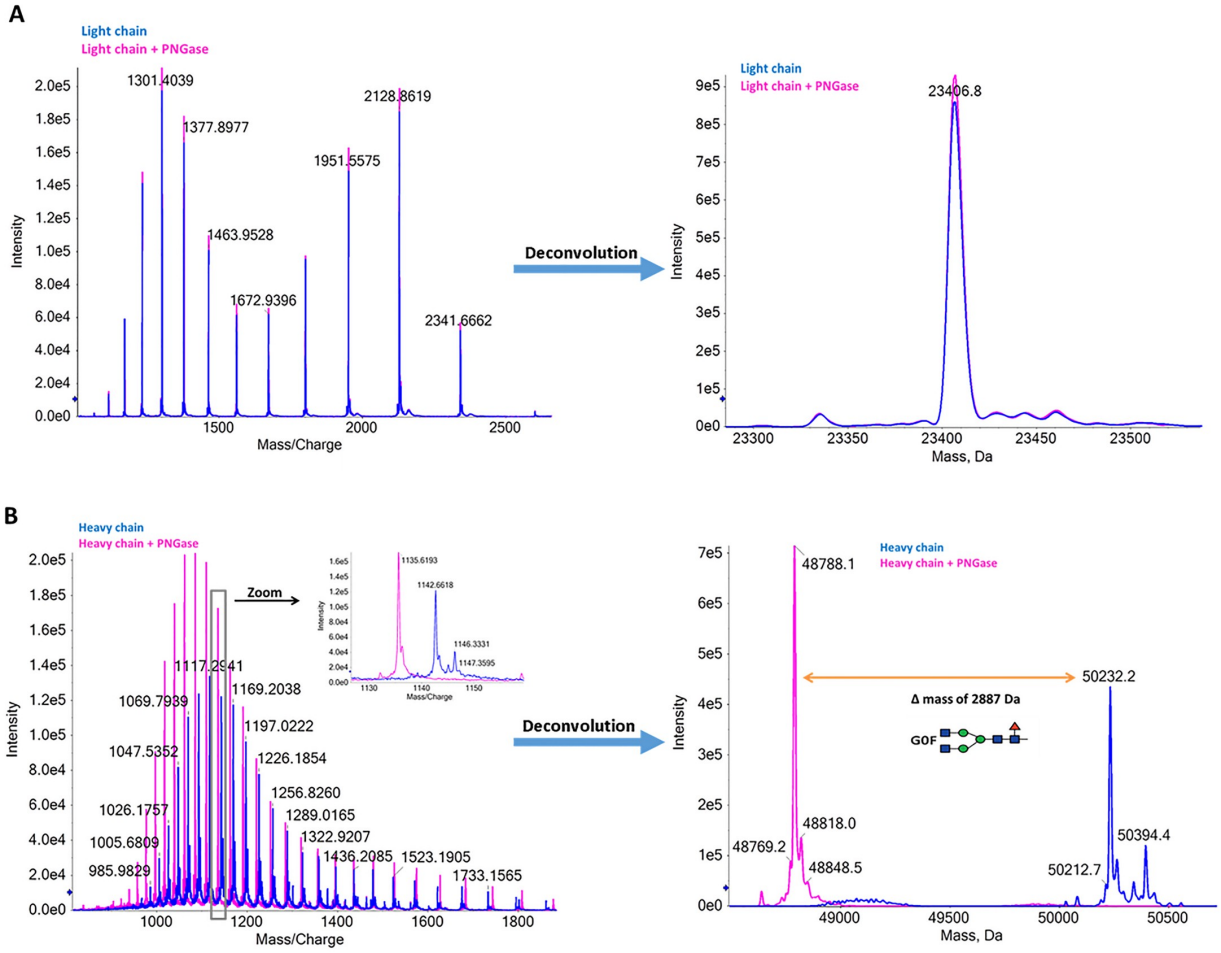
Protein level analysis of reduced monoclonal antibody (characterization level 2). The reduced antibody is shown in the blue and PNGase-treated reduced antibody in the pink. (A) TOF MS spectra (left panel) and deconvolution result (right panel) for the light chain. No mass differences were observed after PNGase treatment. (B) TOF MS spectra (left panel) and deconvolution result (right panel) for the heavy chain. A clear mass shift and reduction of heterogeneity are observed upon PNGase treatment. The mass difference between the two major peaks allowed the determination of the major glycosylation specie, in agreement with the results obtained for the intact antibody.

MS data obtained from reduced antibody analysis are shown in Fig 3. The light chain is homogeneous with a clean charge envelop, unaffected by PNGase treatment (Fig 3A). The reconstructed mass spectrum shows a dominant species with 23,406.8 Da. We did not detect any main protein isoforms for the light chain (Fig 3A).

The heavy chain is heterogeneous due to the presence of different glycoforms (Fig 3B). Upon PNGase treatment, we detected a reduction in mass and heterogeneity, just as observed for the intact antibody analysis. The mass shift observed upon PNGase treatment for the most intense protein species (1444.1 Da) is consistent with the presence of the glycan G0F (Fig 3B). Using this approach, we identified the glycans G0F, G1F, and G2F attached to the heavy chain with 78.1 ± 0.9%, 21.7 ± 0.8%, and 0.3 ± 0.1% (n = 3) abundance, respectively (Fig 3B). These results are in agreement with the intact antibody analysis.

Based on theoretical light chain mass, we obtained a mass difference of -6.24 Da in the reduced analysis. Thus, we can exclude the presence of N-terminal glutamate modification to pyroglutamic acid. It has been described that the inter-chain S-S bonds are more susceptible to chemical reduction than the intra-chain bonds[22]. In this work, we used a DTT-reduction protocol that leads to inter-chain S-S bond cleavage leaving the intra-chain S-S bonds intact. Therefore, we conclude that the light is unmodified (mass error of 2.1 Da). Likewise, from this analysis, we conclude that the heavy chain is modified at the N-terminal with a glutamine modification to pyroglutamic acid (mass error of 0.1 Da for the GOF glycosylated heavy chain).

### 3) Peptide level analysis of tryptic digested antibody

**Fig 4:**
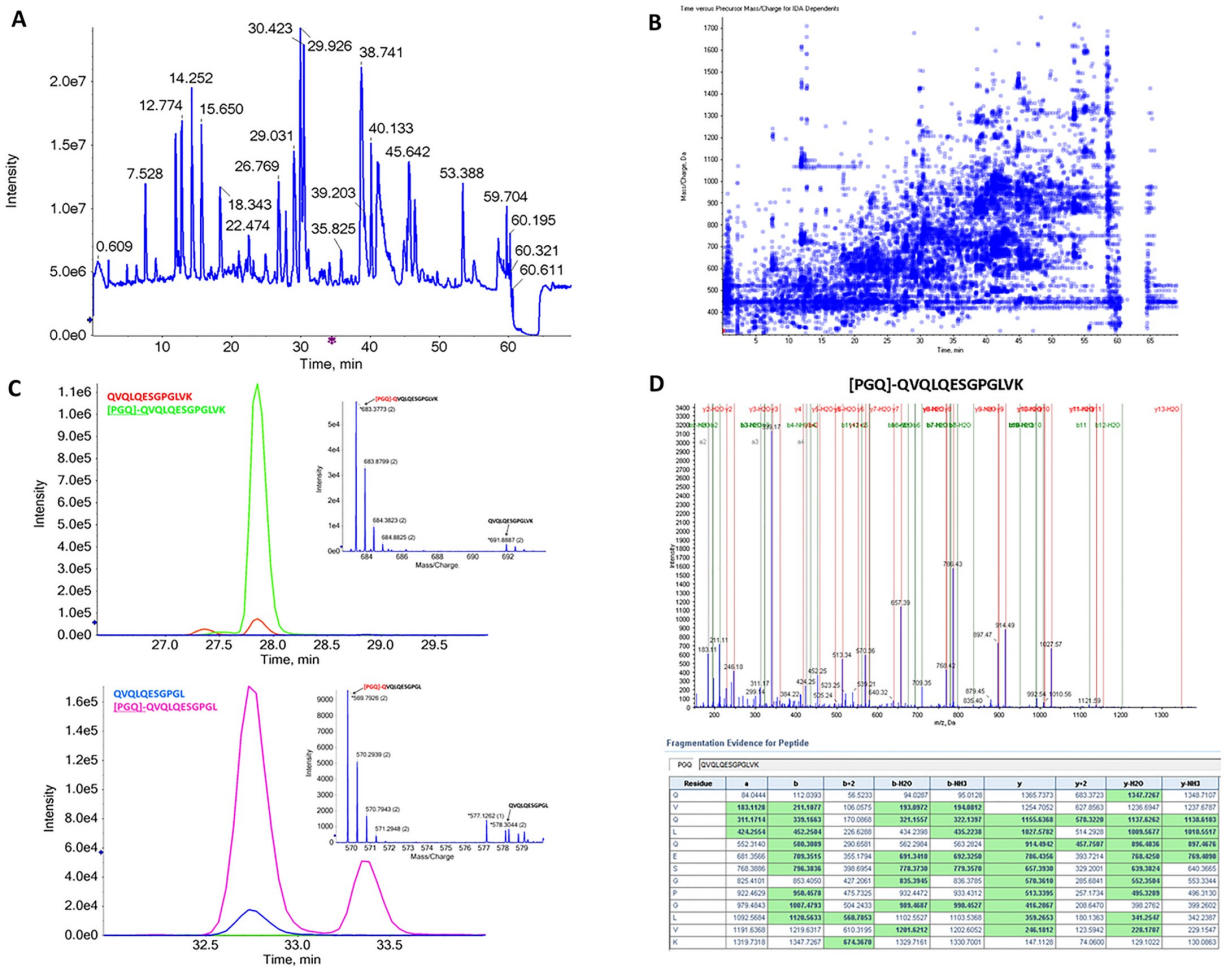
Peptide level analysis of trypsin digested monoclonal antibody (characterization level 3). (A) Total ion chromatogram of 2 μg trypsin digested mAb analysed in microLC-MSMS. (B) IDA dependents profile showing the distribution of precursor m/z values over time. (C) Extracted ion chromatograms (XICs) of heavy chain N-terminal Q modifications. The inset shows the respective mass spectrum. (D) MSMS data of the modified N-terminal peptide ^PGQ^QVQLQESGPGLVK. The green boxes highlighted in the table corresponds to the assigned fragment ions.

The peptide level analysis allows the detection of low abundance protein modifications and the identification of the modified amino acids[16]. The total ion chromatogram obtained from the injection of 2 μg of trypsin-digested antibody is displayed in Fig 4A. The IDA dependents (acquired MSMS spectra distribution in time and m/z range) are showed (Fig 4B). A high abundance of ions over the m/z values range were selected for MSMS analysis, increasing the possibility of obtaining a greater sequence coverage of the protein that allows the detection and identification of peptide modifications. For peptide identifications, we used the ProteinPilotsoftware (v5.0 ABSciex) with a two-entry database consisting of the heavy and light chain amino acid sequences with thorough identification effort. We obtained a full amino acid sequence coverage for the light chain and 98% sequence coverage for the heavy chain (data not shown).

The N-terminal glutamine modification to pyroglutamic acid of the heavy chain, inferred from the protein level analysis of reduced antibody, was unequivocally confirmed using MSMS data at the peptide level (Fig 4D). Using extracted ion chromatograms (XICs) we determined that 95% of the heavy chain N-terminal has this modification (Fig 4C). It is interesting to notice that we can detect a low level of N-terminal glutamate modification to pyroglutamic acid of the light chain when the analysis is done at the peptide level while at the protein level, we could not. This is probably because at the protein level we lack the necessary resolution to detect proteoforms, when they exist in lower abundance. In fact, using extracted ion data we assessed that 99% of the light chain N-terminal is unmodified. Thus, an additional analysis of the antibody at the peptide level should be added to an analytical pipeline as it improves the sensitivity to low abundant modifications. Using this analysis, we also identified and relatively quantified the most frequently reported modifications (asparagine and glutamine deamidation and oxidation/dioxidation products). The results are summarized in Table 1. Results from a representative experiment are shown.

**Table 1:** mAb post-translational modifications detected by peptide mapping analysis with the number of modified peptides identified and the relative abundance of the modification. Results from a representative experiment are shown.

Modifications	# modified peptides	Relative abundance (%)
Deamidated N	224	35.8
Deamidated Q	112	7.4
Oxidation P	59	1.8
Dioxidation P	20	0.8
Oxidation W	29	3.4
Dioxidation W	31	2.1
Trp-Kynurenin W	18	1
Dioxidation Y	22	0.7
Oxidation M	5	1.1
Carbamidomethyl N-term	138	3.9
Carbamidomethyl K	75	1.4

**Fig 5:**
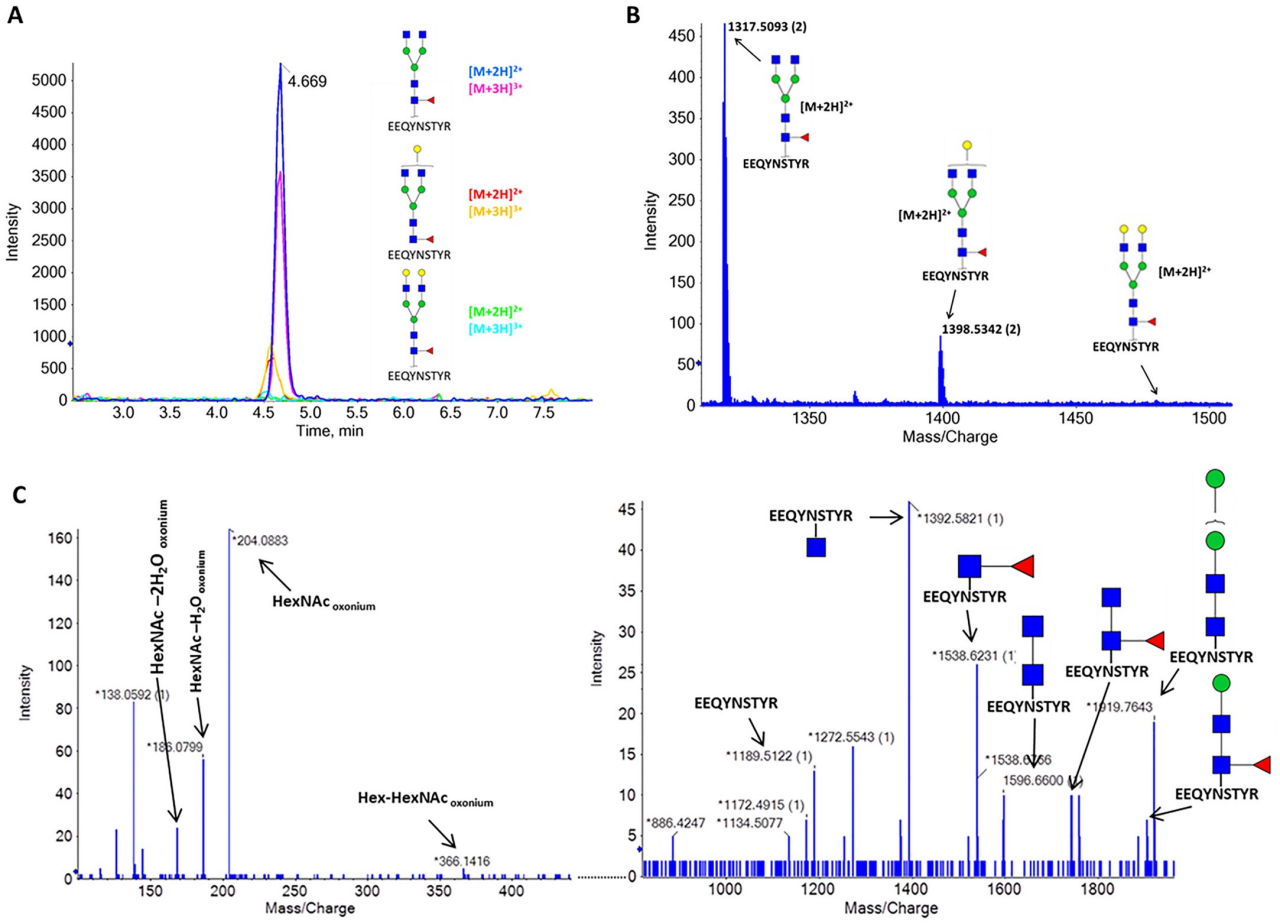
Glycopeptide analysis of tryptic digested monoclonal antibody. (A) XICs of detected glycopeptides. The doubly and triply charged ions were detected for the glycopeptide with the G0F, G1F and G2F glycans structures. (B) Mass spectrum of the doubly charged glycopeptides. (C) MSMS data of the glycopeptide with the G0F structure. Relevant fragments were manually annotated.

One of the most important critical quality attributes of the mAbs is the glycosylation pattern[23,24]. The presence or absence of a glycan structure may have a relevant impact on antibody stability, function, and immunogenicity[23,24]. The glycan composition and pattern are quite diverse, being highly dependent on the mAb expressing conditions[25]. Using the peptide mapping results, we detect three main glycans structures (G0F, G1F, and G2F) attached to the Asn301 on the EEQYNSTYR peptide (Fig 5). Glycopeptides identification was confirmed by MSMS data (Fig 5C). The relative abundances of the different glycan structures agree with the results obtained for the protein level analysis. We also detected the glycan structures G1 and G0F-GlcNac with relative abundance lower than 0.5% (data not shown).

### 4) N-glycan analysis

**Fig 6:**
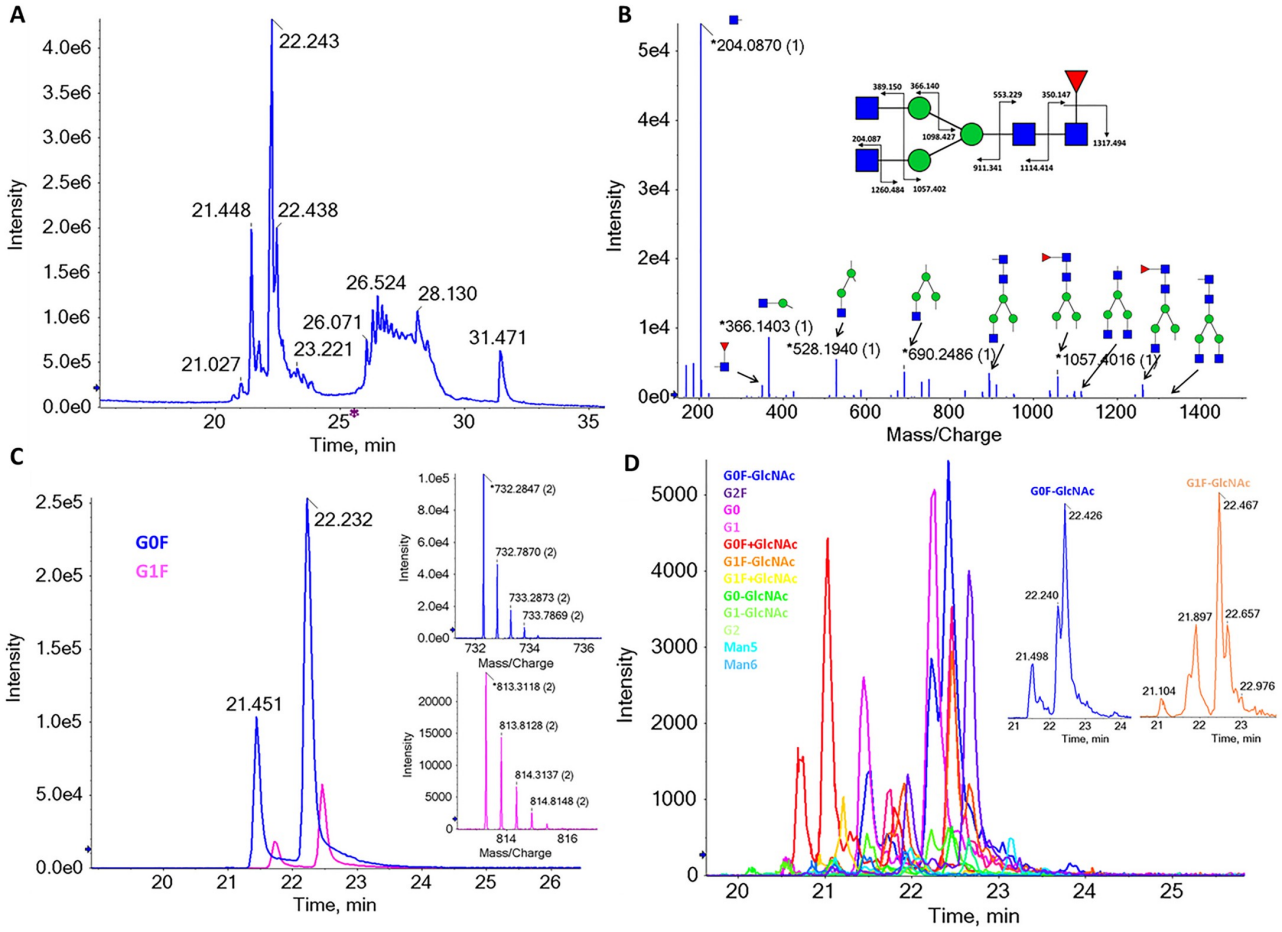
PNGase-released N-glycans analysis (characterization level 4). (A) Total ion current mass chromatogram of N-glycans analysed with PGC nanoLC-MSMS. (B) MSMS data of G0F glycan structure with the higher abundant fragments highlighted. A full analysis of the MSMS data for G0F is shown in supplementary data. (C) XICs of the two most abundant glycan structures, G0F and G1F, and the mass spectra of the corresponding doubly charged m/z ions of these structures. (D) XICs of all other detected glycans. A zoom view of G0F-GlcNAc and G1F-GlcNAc is also shown.

**Fig 7:**
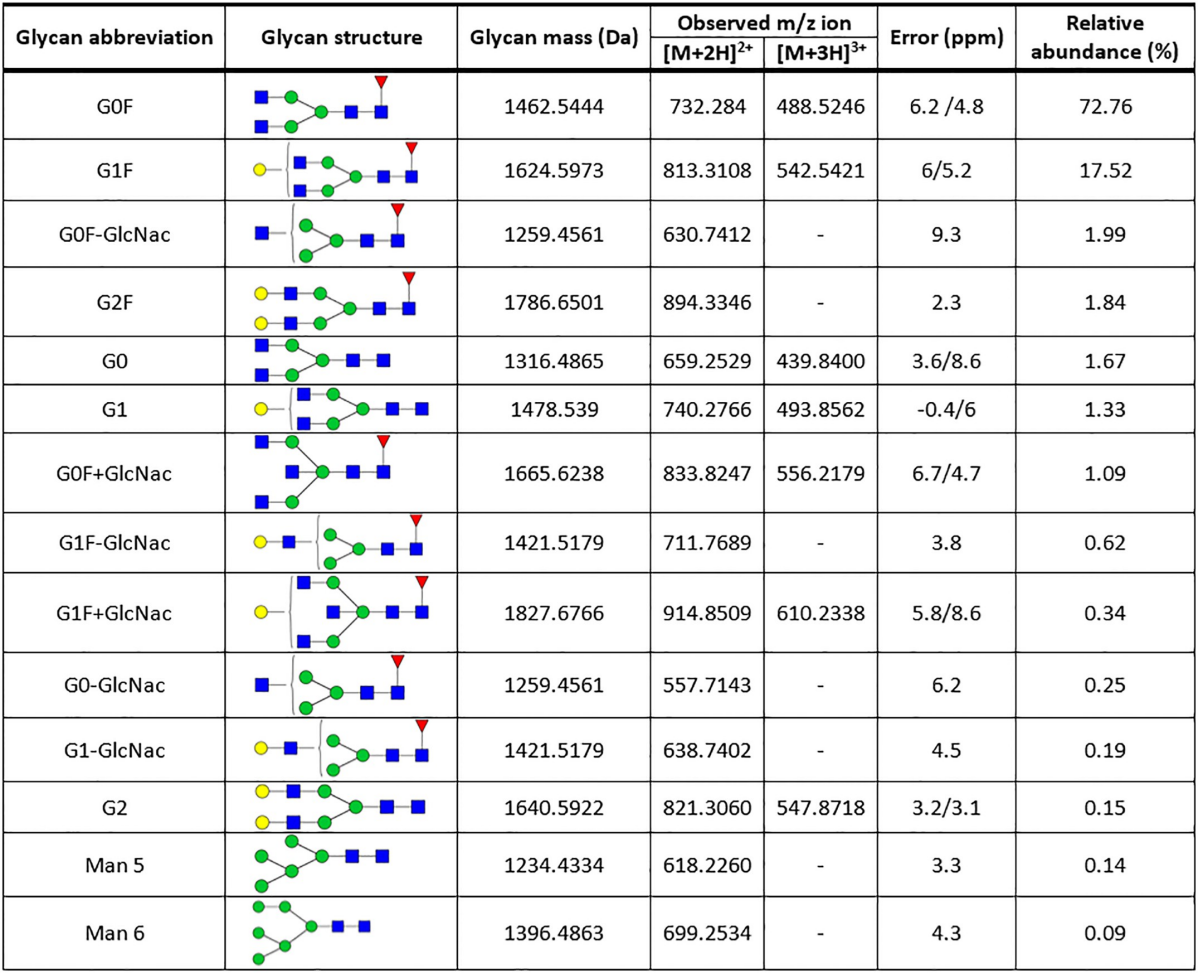
Glycans structures detected in the free N-glycan analysis with the determination of the relative abundance. Results from a representative experiment are shown.

To further confirm the described glycosylation pattern and identify low abundance glycans, we analyzed the N-glycans released by PNGase treatment, using the same MS platform. To increase the assay sensitivity, we performed the analysis by NanoLC-MSMS using porous graphitic carbon (PGC) column. PGC column separates both neutral and sialylated glycans and has the advantage of separating linkage and other structural isomers[26,27]. The total ion chromatogram is shown in Fig 6A. For glycans identification, monoisotopic masses of the molecular ions with the glycan-specific fragment marker ions were searched using the GlycoMod tool from ExPASy. For this analysis, a maximum of 10 ppm mass deviation was considered. Assigned glycan compositions were confirmed by MSMS data using the GlycoworkBench software[21] (Fig 6B and S1 Table). We identified a total of 14 individual glycan structures (summarized in Fig 7). We performed a relative quantification of the detected glycans, using XICs peak areas. Consistently with the results described above, the G0F (73%) and G1F (18%) account for more than 90% of the glycans structures detected (Fig 6C). The remaining glycans detected represent diverse low abundant glycans structures (Fig 7). These low abundance values may explain why these glycans were not detected in the glycopeptide and intact mass analyses. In agreement with previously reported glycan analysis using PGC columns[26], we detected several isomers, as observed in the XICs data. We detected two isomers for the high abundant G0F and G1F (Fig 6C), 4 isomers for the G0F-GlcNAc (Fig 6D inset blue line) and 5 isomers for the G1F-GlcNAc (Fig 6D inset pink line). A detailed description and identification of these glycans isomers will be reported elsewhere.

**Fig 8:**
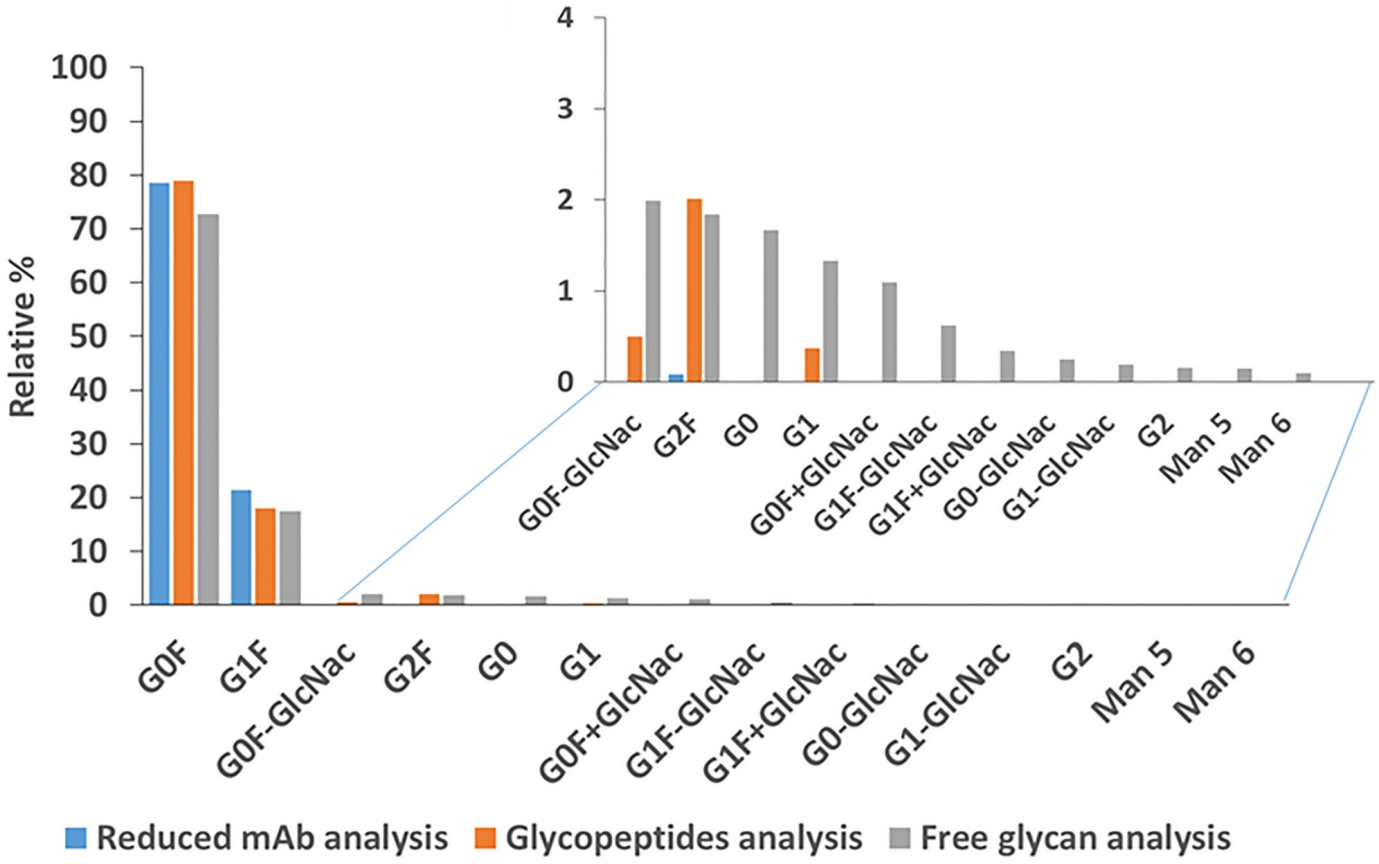
Relative glycosylation quantification using the MS data of the reduced mAb analysis, glycopeptide analysis and free glycan analysis. For the higher abundant glycans structures (G0F and G1F) similar results were obtained. Diverse low abundant glycan structures were only detected in the free glycans analysis.

With these data, we performed a comparison of the glycosylation relative quantification using the data of the different characterization levels (intact mass, glycopeptides, and free N-glycans analysis) (Fig 8). Of interest, we obtained similar glycosylation abundance for the major glycans structures (G0F and G1F). However, we only detect low abundant glycan structures (total relative abundance lower than 2%) in the N-glycan analysis using Nano-LCMSMS. This means that major differences in the glycosylation profile of different mAb candidates can be screened efficiently and straightforward by intact mass analysis as described here. Nonetheless, a detailed characterization of mAbs microheterogeneity (glycans abundances lower than 2%) needs further investigation and different levels of analysis (as demonstrated by glycopeptides and free N-glycan analysis).

## Conclusion

Using a general and simplified mAbs preparation strategy (protein desalting using amicon devices; protein reduction; PNGase-F N-glycan removal and trypsin digestion), an extensive MS-based characterization of a mAb molecule is presented. This method allowed the confirmation of mAbs molecular mass, the assessment of glycosylation and micro-heterogeneity, as well as the detection of other PTMs as deamidation of asparagine, isomerization of aspartate, oxidation of methionine and tryptophan and variability of N- and C-terminus of the polypeptide chain. This multi-attribute method was performed using a single MS-platform for the analysis of intact protein mass, (glyco)peptide mapping and released N-glycans, providing semi-quantitative data. Noteworthy, we used a general sample preparation and MS workflow, without the requirement of extensive method development. Thus, instead of using an array of separation techniques and analytical tools, the presented workflow allows the simultaneous detection, identification, and semi-quantification of several product properties (at the molecular level) using a single analytical platform.

## Supporting information

S1 Table Detailed analysis of the MSMS data for G0F. (DOCX)

## Abbreviations

DTT: dithiothreitol
ESI: electrospray ionization
G0F: glycan (GlcNAc)2Man3(Fuc)(GlcNAc)2
G1F: glycan Gal-(GlcNAc)2Man3(Fuc)(GlcNAc)2
G2F: glycan Gal2-(GlcNAc)2Man3(Fuc)(GlcNAc)2
GOF—GlcNAc: glycan (GlcNAc)2Man3(Fuc)GlcNAc
Hex: Hexose
HexNAc: N-Acetylglucosamine
ID: Inner diameter
IDA: Information dependent analysis
LC: Liquid chromatography
LC-MS: Liquid chromatography mass spectrometry
LC-MSMS: Liquid chromatography tandem mass spectrometry
mAbs: monoclonal antibodies
MS: Mass spectrometry
MSMS: tandem mass spectrometry
PGC: porous graphitic carbon
PNGase: Peptide:N-glycosidase F
PTM: Post-translational modifications
QTOF MS: Quadrupole time-of-flight mass spectrometry
TIC: Total ion chromatogram
TOF MS: Time-of-flight mass spectrometry
XIC: Extracted ion chromatogram
