# Supplementary figures and images for: Exploring the analytical power of the QTOF MS platform to assess monoclonal antibodies quality attributes
Source: PLoS One. 2019 Jul 10;14(7):e0219156. doi: 10.1371/journal.pone.0219156 (PMC6619757; doi:10.1371/journal.pone.0219156)

Supplementary table 1 – Detailed analysis of the MSMS data for G0F.

| Fragment [M+H+]+ | Structure |
| --- | --- |
| 1260.4843 | 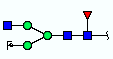 |
| 1242.4739 | 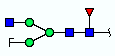 |
| 1114.4218 | 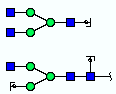 |
| 1098.4273 | 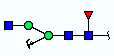 |
| 1096.4122 | 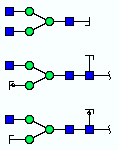 |
| 1080.4220 | 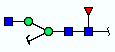 |
| 1057.4016 | 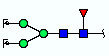 |
| 1039.3905 | 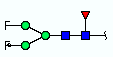 |
| 952.3686 | 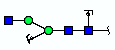 |
| 934.3595 | 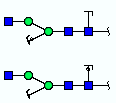 |
| 911.3414 | 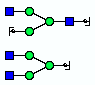 |
| 893.3299 | 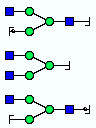 |
| 877.3344 | 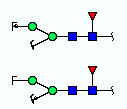 |
| 749.2837 | 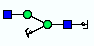 |
| 732.7883 | 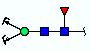 |
| 731.2753 | 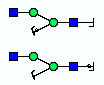 |
| 715.2759 | 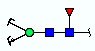 |
| 708.2592 | 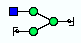 |
| 690.2486 | 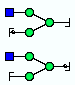 |
| 571.2373 | 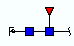 |
| 553.2289 | 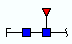 |
| 546.2065 | 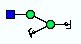 |
| 528.1940 | 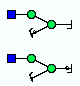 |
| 510.1860 | 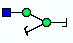 |
| 425.1766 | 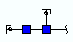 |
| 407.1677 | 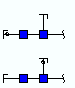 |
| 389.1504 | 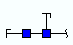 |
| 384.1505 | 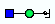 |
| 366.1403 | 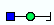 |
| 350.1457 | 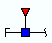 |
| 222.0975 | 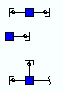 |
| 204.0870 | 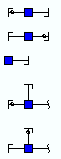 |
| 186.0762 | 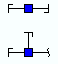 |

Supplement: S1 Table — (DOCX) [file pone.0219156.s001.docx]
